# Temperature and Diet Acclimation Modify the Acute Thermal Performance of the Largest Extant Amphibian

**DOI:** 10.3390/ani12040531

**Published:** 2022-02-21

**Authors:** Chun-Lin Zhao, Tian Zhao, Jian-Yi Feng, Li-Ming Chang, Pu-Yang Zheng, Shi-Jian Fu, Xiu-Ming Li, Bi-Song Yue, Jian-Ping Jiang, Wei Zhu

**Affiliations:** 1 Key Laboratory of Bioresources and Ecoenvironment (Ministry of Education), College of Life Sciences, Sichuan University, Chengdu 610064, China; zhaocl_1@163.com; 2 CAS Key Laboratory of Mountain Ecological Restoration and Bioresource Utilization & Ecological Restoration Biodiversity Conservation Key Laboratory of Sichuan Province, Chengdu Institute of Biology, Chengdu 610041, China; zhaotian@cib.ac.cn (T.Z.); fengjy@cib.ac.cn (J.-Y.F.); changliming16@mails.ucas.ac.cn (L.-M.C.); zhengpy@cib.ac.cn (P.-Y.Z.); jiangjp@cib.ac.cn (J.-P.J.); 3 Laboratory of Evolutionary Physiology and Behavior, Chongqing Key Laboratory of Animal Biology, Chongqing Normal University, Chongqing 400047, China; shijianfu9@hotmail.com (S.-J.F.); xiumingli418@hotmail.com (X.-M.L.)

**Keywords:** *Andrias davidianus*, animal conservation, metabolic compensation, physiological plasticity, respiration rate, thermal limits

## Abstract

**Simple Summary:**

The Chinese giant salamander (*Andrias davidianus*) is one of the largest extant amphibian species, and it is considered critically endangered by the IUCN Red List. Previous studies have demonstrated that future climate change could strongly affect this species. However, how to conduct the related conservation activities are still unclear. Understanding the thermal physiology of *A. davidianus* is meaningful in guiding its conservation, e.g., habitat selection and preadaptation before population translocation. In this study, the influences of temperature and diet on the metabolic capacity and thermal limits were studied for *A. davidianus* larvae based on laboratory experiments. Our results indicated prominent physiological plasticity in the thermal tolerance of *A. davidianus* in response to temperature and diet changes. This thermal plasticity likely, to some extent, buffers the effects of climate change on the Chinese giant salamander. In addition, the potential mechanisms underlying this plasticity were discussed. Our results provide insights for the formulation of conservation strategies for this species.

**Abstract:**

The Chinese giant salamander (*Andrias davidianus*), one of the largest extant amphibian species, has dramatically declined in the wild. As an ectotherm, it may be further threatened by climate change. Therefore, understanding the thermal physiology of this species should be the priority to formulate related conservation strategies. In this study, the plasticity in metabolic rate and thermal tolerance limits of *A. davidianus* larvae were studied. Specifically, the larvae were acclimated to three temperature levels (7 °C, cold stress; 15 °C, optimum; and 25 °C, heat stress) and two diet items (red worm or fish fray) for 20 days. Our results indicated that cold-acclimated larvae showed increased metabolic capacity, while warm-acclimated larvae showed a decrease in metabolic capacity. These results suggested the existence of thermal compensation. Moreover, the thermal tolerance windows of cold-acclimated and warm-acclimated larvae shifted to cooler and hotter ranges, respectively. Metabolic capacity is not affected by diet but fish-fed larvae showed superiority in both cold and heat tolerance, potentially due to the input of greater nutrient loads. Overall, our results suggested a plastic thermal tolerance of *A. davidianus* in response to temperature and diet variations. These results are meaningful in guiding the conservation of this species.

## 1. Introduction

Temperature is one of the most important climatic factors for ectotherms. Variations of environmental temperatures can not only impact the fitness of ectotherms directly by reducing the behavior and physiological performance [1], but they also disrupt the homeostasis of ecosystems and cause the spread of pathogens or shrink of habitat [2,3]. The critical thermal limit (T_c_, including upper T_c_ and lower T_c_) is the most widely used index representing the thermal tolerance of animals [4,5]. It is defined as the thermal point at which locomotory activity becomes disorganized and the animal loses its ability to escape from conditions in gradually heating or cooling thermal conditions [6]. Many studies suggested that the T_c_ are linked to species geographical distributions by matching with the local climate [7,8,9,10], making it an important index in predicting the population dynamics of animals under climate change [11,12]. However, the biological processes that determine the thermal tolerances of animals are still controversial. One of the best-known hypotheses is the “Oxygen Limitation of Thermal Tolerance” [13], which claims that a mismatch between the demand for oxygen and the capacity of oxygen supply to tissues is the critical mechanism restricting the whole animal’s tolerance to thermal extremes [14]. The association between aerobic metabolism and upper T_c_ has been supported by a large body of evidence from fish [15] and arthropods [16]. Moreover, a higher metabolic rate is always associated with better locomotory performance under cold stress [17,18]. These results suggest that the metabolic architecture of animals can provide mechanistic insight into their thermal tolerance.

The thermal tolerances of many ectotherms are plastic with the change of the environment. These animals can remodel their cellular processes and structure to reduce the variation degree of physiological activities in response to environmental changes [19]. For example, ant foragers in late summer had higher average upper T_c_ compared to those in March and December [20]. More evidence is provided from laboratory studies [21,22]. This physiological plasticity is called acclimation or acclimatization capacity. From a metabolic perspective, thermal acclimation induces metabolic compensation in individuals to offset the thermodynamic variations in metabolic reactions [23]. Plasticity enabled by thermal acclimation is expected to broaden the range of temperatures at which animals are active [24] and is often highlighted as a powerful mechanism to buffer the impact of climate change [25,26]. In addition to environmental temperature, variations in other factors (e.g., oxygen level, water level, and nutrient conditions) can also affect the thermal tolerance (called the cross-talk effect) [27,28]. Therefore, it is necessary to consider the plasticity in thermal tolerance at multiple conditions to better understand species’ thermal physiology.

Global decline in the amphibian populations is an urgent environmental and ecological problem [29]. The Chinese giant salamander (*Andrias davidianus*) is one of the largest extant amphibian species, and it is often referred to as a living fossil [30]. This species was once widely distributed in central and southern China [31,32]. In the past 60 years, the wild populations of *A. davidianus* have declined dramatically due to habitat degradation, pollution, and overexploitation [33,34]. Recently, this species was evaluated as critically endangered by the International Union for Conservation of Nature Red list [33] and Chinese specialists [35]. Its evolutionary and ecological significance makes it a flagship species for biodiversity conservation in China. In particular, the effect of climate change (e.g., rise of temperature) has been considered a severe challenge to its survival in the wild [34,36]. In addition to the climatic factors, empirical evidence from farmers and our laboratory indicate that the diet types (e.g., fish, red worm, and pork liver in the farming industry) and temperature can jointly affect the growth rate, a primary fitness index, of the *A. davidianus* larvae. It is interesting to know whether the diet type can shape the thermal physiology of these animals. This knowledge can be implicative in their conservation in the context of climate change.

In this study, the influences of acclimation temperature and diet on the thermal performance (i.e., metabolic capacity and thermal limits) were studied for the Chinese giant salamander larvae. Our main target was to study the plasticity in the thermal performance of this species. This knowledge may extend our understanding of the physiology of this important species and provide useful information for the conservation of the wild *A. davidianus* population under future climate change.

## 2. Materials and Methods

### 2.1. Animals and Acclimation

Larvae of *A. davidianus* from the same clutch were purchased. They were cultured under the same environmental conditions in a farm located at Hongya, Sichuan Province, China (103°10′05″ E, 29°52′36″ N). Specifically, the average water temperature was 15 (±1.1 SD) °C, and their main food throughout the first year after hatching was red worm. Once collected from the farm, the larvae were cultured in artificial rearing tanks (length × width × height = 29 cm × 20 cm × 9.7 cm; with 3000 mL water) in laboratory conditions (light: dark = 12: 12; 15 ± 0.5 °C; dissolved oxygen level >90%) for two weeks before treatments. The larvae were fed with sufficient red worm twice a day at 09:00 a.m. and 18:00 p.m., respectively. Water was replaced daily. Animal procedures were approved by the Animal Care and Use Committee of the Chengdu Institute of Biology, Chinese Academy of Sciences.

According to laboratory studies, *A. davidianus* larvae are sensitive to temperature variations [37,38]. The optimal growth occurs in water with a temperature range of 15–21 °C [39]. Their feeding behavior was inhibited by water temperature higher than 25 °C or lower than 8 °C [40,41]. Therefore, three discrete temperature levels (7 °C/cold stress, 15 °C/optimum, and 25 °C/heat stress) were selected for thermal acclimation. The red worm (*Limnodrilus* sp.) was an empirical diet for captive giant salamanders at the first year after hatching, while wild individuals likely have more diverse prey, including fish fray, frogs, and arthropods. In this study, the red-worm and fish fray were selected to feed the experimental larvae.

**Figure 1:**
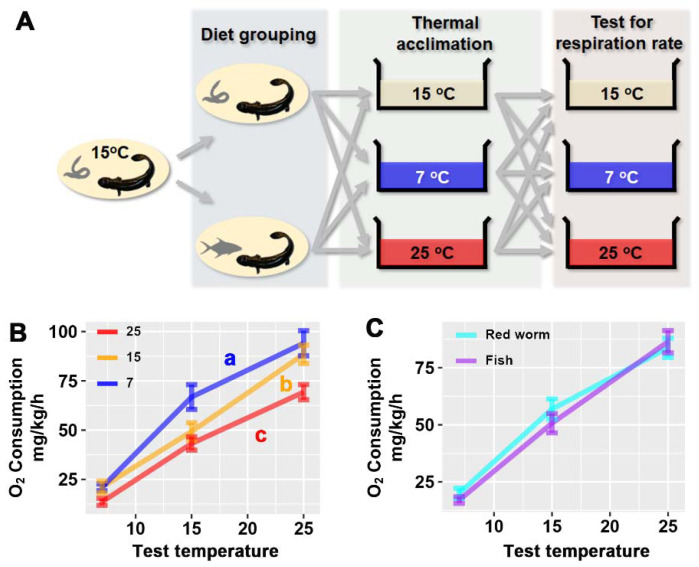
Plasticity in metabolic capacity after thermal and diet acclimation. (**A**) Experimental design. (**B**) Variations between thermal-acclimated groups. (**C**) Variations between diet-acclimated groups. The data were analyzed by ANCOVA (test temperature as covariant) and LSD post hoc test; the results are detailed in Table 2. Different letters denote significant differences between groups.

**Figure 2:**
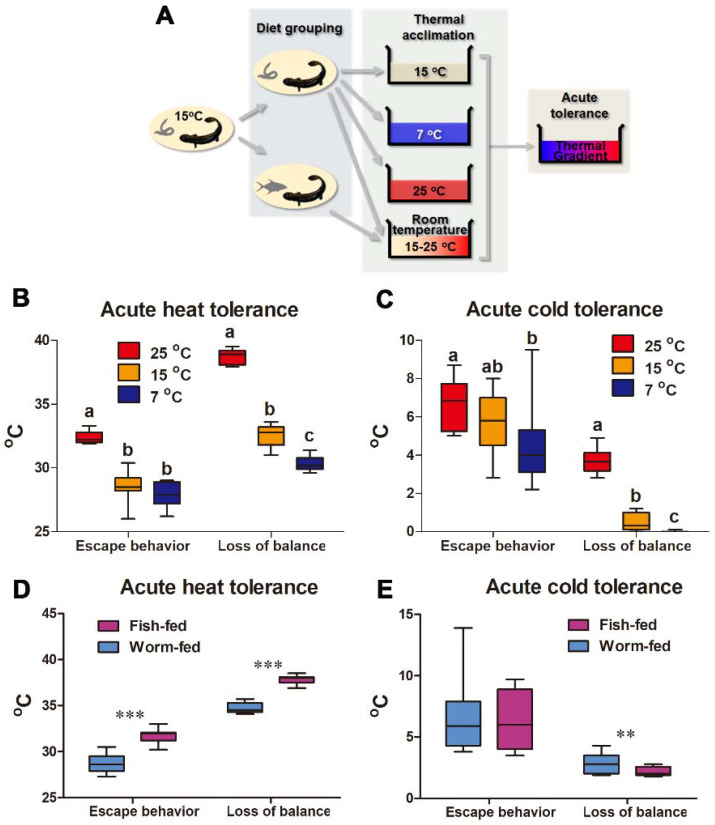
Plasticity in thermal limits after thermal and diet acclimation. (**A**) Experimental design. (**B**,**C**) Variations between thermal-acclimated groups. Different letters denote significant differences (*p* < 0.05) between groups; Kruskal–Wallis test. (**D**,**E**) Variations between diet-acclimated groups. The differences between worm and fish diets were analyzed by Mann–Whitney U test; **, *p* < 0.01; ***, *p* < 0.001.

Two independent acclimation programs were conducted successively to measure the respiration rate and thermal limits, respectively. For the measurement of respiration rate, 210 larvae were collected on their 60th day after hatching. After two weeks of laboratory acclimation, these larvae (1.65 ± 0.3 g, mean ± SD) were randomly divided into six groups: worm diet at 7 °C, worm diet at 15 °C, worm diet at 25 °C, fish diet at 7 °C, fish diet at 15 °C, and fish diet at 25 °C (Figure 1A). Each group included two tanks, and each tank kept 15–20 individuals. The nutrient composition of red worm and fish fray is presented in Table 1. The whole acclimation duration lasted for 20 days. The daily culture followed the conditions described above. The body weight of the larvae was measured at the nearest 0.01 g at the end of treatment, as well as before the measurement of respirate rate. To measure thermal limits, 150 larvae were collected from the farm on their 120th day after hatching. After two weeks of laboratory acclimation, these larvae (3.26 ± 0.5 g, mean ± SD) were randomly divided into five groups (30 individuals for each group): worm diet at 7 °C, worm diet at 15 °C, worm diet at 25 °C, worm diet at room temperature (15–25 °C), and fish diet at room temperature (15–25 °C) (Figure 2A). The whole acclimation duration lasted 30 days. For worm diet at 7 °C, worm diet at 15 °C, worm diet at room temperature (15–25 °C), and fish diet at room temperature (15–25 °C) acclimation groups, 15 individuals were tested for either upper or lower thermal limits. However, only 10 individuals were tested at the lower limit for worm diet in the 25 °C-acclimated group as 5 individuals died during the acclimation.

### 2.2. Respiration Rate

Oxygen consumption of *A. davidianus* individuals was measured by intermittent respirometer. For each round of tests, the respiration rate of one larva was measured. All individuals were fasted for 24 h before measurement, and the animal could swim freely in the chamber. For any acclimation groups, 11–18 individuals were randomly selected and measured at each test temperature (i.e., 7, 15, or 25 °C). For each individual, the respiration rates at 7, 15, and 25 °C were not measured continuously. Instead, these measurements were separated by recovery intervals (24 h) at the acclimation temperatures to make sure the larvae returned to their initial status. During the recovery intervals, the larvae were placed back in their respective tanks. It should be noted that the larvae from the same tank (more than one individual in each tank) could not be distinguished from each other, as there was no suitable method to label them. This meant that the same larva might be selected to measure the respiration rate at two or three different test temperatures. Thus, to avoid pseudo-replication in the statistical models, the average value of the respiration measurements, which shared the same test temperature, acclimation temperature, and diet type (n = 11–18 individuals or measurements for each condition), was treated as the only replication for each condition. See the raw data in Supplementary files.

The measurement began when the larvae became quiet. During measurement, their movements, if any, were transient and limited in small ranges as the chamber is small, and most of the time, these larvae were stationary. The respirometer consisted of one chamber (0.15 L) and one dissolved oxygen analyzer (HQ30d, HACH company, New York, USA). In addition, the intermittent respirometer was equipped with an internal circulation pump to fully mix the dissolved oxygen in the internal water. One chamber in each group without *A. davidianus* individual was used as a blank control chamber to calculate the background oxygen consumption. Then, oxygen consumption rate (VO_2_) was measured one time at 1 min intervals for 30 min. The following formula was used to calculate VO_2_ (mg O_2_kg^−1^h^−1^) [42]:

$${\mathrm{VO}}_{2}=\Delta O_{2}\times v/m$$

where $\Delta O_{2}$ is the difference in the oxygen concentration level (mg O_2_ L^−1^) between the experimental and blank control chamber, v is the water flow rate in the chamber (Lh^−1^), and m is the body weight of the *A. davidianus* individual (kg).

### 2.3. Measurement of Thermal Limits

The thermal limits of the larvae were measured after acclimation. The larvae were placed in a digital water bath, and a square space (height × width × depth = 23 × 11 × 18 cm) was delimited by a plastic net to avoid the direct touch between animals and the bath. The water temperature was initially set at 20 °C, and it increased or decreased by ~1 °C/min. The larvae exhibited free swimming at the beginning and then exhibited behavioral agitation (struggled to escape the confinement). The water temperature for the onset of escaping behavior was recorded, and it was defined as the escaping temperature (T_esp_). Once the larvae exhibited escaping behavior, they were turned over by a glass hook every 1 min. The water temperature was considered to have reached the critical temperature (T_c_) once the larvae exhibited a loss of righting response (five seconds). The loss of righting response was characterized by the animal’s inability to correct their orientation after being flipped upside down using a glass rod, while submerged in water [43]. Note that the absolute Tc values might be overestimated, as there was likely a lag in the variation of the body temperature following the variation of the water temperature. Each individual was tested for either lower thermal limits or upper thermal limits.

### 2.4. Statistical Analyses

Statistical analyses were conducted on SPSS v25.0 (SPSS Inc., Chicago, IL, USA). The differences in growth rate between groups were analyzed by ANOVA, with acclimation temperature and diet as two independent factors. The variations in respiration rates were analyzed by ANCOVA and LSD post hoc test, with test temperature as a covariant. The interactive effects between the independent factors were considered in the ANOVA and ANCOVA models. The differences in upper or lower T_c_ and T_esp_ were analyzed by Kruskal–Wallis or Mann–Whitney U tests. Graphs were generated by Graphpad Prism 5 or ggplot2, an R package [44].

## 3. Results

The interaction between environmental temperature and diet affects the larvae growth rate (F_2,87_ = 20.356, *p* < 0.01, two-way ANOVA; Table S1). In worm-fed groups, 7 °C larvae had decreased somatic growth compared to their 15 °C counterparts. However, no significant acceleration in growth was detected between larvae from the 7 °C group and those from the 25 °C group (simple effect analysis; Figure S1). In fish-fed groups, the growth rate tended to increase with the rise of environmental temperature, but the inter-group variations were not significant (simple effect analysis; Figure S1).

### 3.1. Influences of Temperature and Diet Acclimation on Larvae Metabolism

Acclimation temperature affect the larvae’s metabolic rate (presented as oxygen consumption rate) independently (F_2,11_ = 10.987, *p* = 0.002; Table 2). Cold acclimation enhanced the metabolic capacity of larvae, while warm acclimation reduced their metabolic capacity (*p* < 0.05, LSD post hoc test; Figure 1B). The larvae metabolism was not significantly affected by diet (F_1,11_ = 0.468, *p* = 0.508; Figure 1C).

### 3.2. Influences of Temperature and Diet Acclimation on the Acute Thermal Tolerance Window

The influences of acclimation temperature and diet on acute thermal tolerance were studied (Figure 2A). Temperature acclimation caused a shift of the acute thermal tolerance windows of giant salamanders (Figure 2B,C). The upper T_esp_ and T_c_ of warm-acclimated larvae increased from 28.57 ± 1.2 °C and 32.52 ± 0.8 °C to 32.39 ± 0.5 °C and 38.72 ± 0.6 °C, respectively (mean ± SD, and similarly hereinafter). This was accompanied by an increment in their lower T_esp_ and T_c_ from 5.51 ± 1.5 °C and 0.48 ± 0.4 °C to 6.63 ± 1.3 °C and 3.72 ± 0.7 °C, respectively. Cold-acclimation caused opposite changes, with their lower T_esp_ and T_c_ decreasing to 4.64 ± 2.1 °C and 0 ± 0.02 °C, respectively, which was accompanied by a decrease in the upper T_esp_ and T_c_. Fish-fed larvae exhibited a wider thermal tolerance window than worm-fed individuals, characterized by increased upper T_esp_ and T_c_ and decreased lower T_c_ (Figure 2D,E).

## 4. Discussion

### 4.1. Temperature-Induced Shift of Thermal Limits

Our results indicated that acclimating *A. davidianus* larvae to higher and lower environmental temperatures decreased and increased their metabolic capacity, respectively, suggesting metabolic compensation and adaptive plasticity in response to thermal fluctuation. Thermal compensation enables the maintenance of physiological rates across environmental conditions. This was frequently observed in animals facing mildly cold environments [23,45,46]. This biological phenomenon is believed to partly offset the reduced physiological performance under cold and thus allows better exploitation of the environment by maintaining physiological activities such as locomotion, feeding, and development at low temperatures [47]. For animals in warming conditions, their increased metabolic activity due to thermodynamic effects resulted in accelerated resource consumption. Downregulation of metabolism can benefit their long-term survival from the perspective of resource-saving [48,49,50]. Given that amphibians’ larval stage is devoted to somatic growth and energy storage [51], either an increased metabolic rate at a lower temperature or decreased metabolic rate at a higher temperature are aligned with their life strategy.

This study measured the thermal limits of *A. davidianus* larvae in the early developmental stage. It demonstrated their plasticity in thermal tolerance. The ability to acclimatize to changing thermal conditions is expected to be a primary factor that dictates the vulnerability of taxa to climate change. Generally speaking, the plasticity in thermal tolerance means better outcomes for *A. davidianus* larvae in response to global warming or extreme weather than expected. Currently, the existence and distribution of wild *A. davidianus* in the future has become a topic that is in great need of research and discernment. The ecological niche model raised by Zhao, et al. [34] suggested that climate change can potentially affect the distribution of these animals. To have a more reliable and refined prediction of the fate of the wild populations, physiological models considering their thermal limits and plasticity should be considered. Our results may provide a foundation for these studies. However, two factors should be still be considered, the variation of thermal tolerance with life stages [4] and the potential differences in thermal traits between different phylogenic clades of *A. davidianus* [52]. Further studies should focus on these questions, particularly in clarifying which life stage or phylogenic clades exhibit the lowest thermal tolerance and plasticity. This knowledge could be meaningful in guiding the conservation of wild *A. davidianus*, e.g., making more reliable predictions of the individual survival from different geographical populations and optimizing the trophic structure of their habitats to enhance their tolerance to extreme weather conditions.

Making clear the mechanisms underlying the thermal tolerance may guide the conservation of Chinese giant salamander in the context of climate change. Despite the complexity in the mechanisms of acute thermal intolerance, metabolic capacity is always an important contributor to the thermal tolerance of aquatic ectotherms [14,53]. This is particularly true for cold conditions, where the ectotherms have difficulty maintaining their metabolic activities. For example, the *atu* mutant *Drosophila melanogaster* has increased metabolic capacity, which improves its cold tolerance [54]. Accordingly, variations in metabolic capacity explained the improved and weakened cold tolerance in cold- and warm-acclimated individuals, respectively. For heat tolerance, as the activities of most biological reactions increase with ambient temperatures within certain thermal scopes, the metabolic capacity may no longer be a major limiting factor for heat tolerance. Instead, the overactive metabolic capacity can limit heat tolerance by overburdening the oxygen supply [13,14]. Additionally, it was reported that downregulation of metabolism could improve the acute heat tolerance of animals by reducing their requirement for oxygen [55]. Accordingly, decreased metabolic capacity after heat acclimation is likely associated with higher upper limits and vice versa. Taken together, the metabolic rate may be an important factor for predicting the tolerance of *A. davidianus* to extreme temperature. In conservation practice, to improve the survival rate of reintroduced populations, we might screen individuals whose thermal tolerance matches the climatic characteristics of the translocation habitats, e.g., introducing cold-tolerant individuals to regions with severe winters or introducing warm-tolerant individuals to hot environments. As direct measurement of the thermal tolerance is detrimental or even lethal to animals, the metabolic rate can be an applicable indicator for their thermal-tolerance properties.

### 4.2. Diet-Induced Shift of Thermal Limits

Unlike the temperature acclimation, which induced a unidirectional shift of thermal limits, diet acclimation broadened the thermal-tolerance window of *A. davidianus* larvae in both directions. Diet did not change the metabolic capacity of giant salamander. It implied different mechanisms between diet and temperature acclimations in affecting thermal tolerance. Nutrition was reported to modify the critical thermal limits of ectotherms [56,57]. Despite the effects of nutrition depending on the animal size or thermal conditions [58], most studies supported that nutrient supplements (e.g., carbohydrates and amino acids) can improve thermal tolerance in animals [56,59,60,61,62,63,64]. This is reasonable, as rich nutrient storage can improve cellular metabolic maintenance and benefit the synthesis of protectants, which are necessary for survival in stressful conditions. In our study, the most prominent difference between fish and red-worm diets was that the former was richer in total energy, protein, and lipid levels (Table 1). This might be a reason for the superior of fish-fed larvae in thermal tolerance. This finding suggests that the productivity or prey abundance of the natural habitats could be a potential determinator for the thermal tolerance of wild *A. davidianus*. Introducing suitable prey with great nutrient loads to the natural or artificial habitats of *A. davidianus* may be an alternative approach to reduce the impact of climate change and extreme weather on these animals.

Some mechanisms by which the nutrients modify the thermal physiology of animals were revealed. For example, it was reported that the storage of carbohydrates, the anaerobic substrate, is associated with the tolerance of animals to extreme heat stress [16,65] when the oxygen supply cannot match the metabolic requirement [14]. Although the fish diet contains less sugar than the worm diet, the protein level in the former is much higher. Amino acids derived from proteolysis can be easily converted into carbohydrates. For cold tolerance, the abundance of lipids should be important, as these compounds can be major metabolic substrates in cold conditions [47,66]. More importantly, lipids are required for cellular membrane remodeling, which is an important mechanism underlying cold tolerance [67]. Accordingly, the rich lipid in the fish diet might contribute to the cold tolerance of fish-fed larvae. Overall, these findings shed light on the new thoughts referring to the conservation of this species under climate change. Currently, the reintroduction of captive-bred individuals to the historical natural habitats has been an important measure for the recovery of wild giant salamander populations [68]. Since the diet is a determinant of thermal performance, the prey abundance and diversity in the habitat should be considered before reintroduction. Moreover, to improve the survival rate of the released giant salamander, a preadaptation procedure should be conducted before reintroduction.

## 5. Conclusions

In this study, we demonstrated the plasticity in thermal physiology of *A. davidianus* larvae in response to environmental temperature and diet changes. These larvae exhibited apparent thermal compensation in metabolic capacity after thermal acclimation. Specifically, cold- or heat-acclimation improved their tolerance to more extreme thermal stress but compromised their tolerance to the opposite thermal extremes. Diet did not affect the metabolic rate; however, the fish diet, which was richer in protein, lipid, and total energy, broadened the thermal-tolerance window of *A. davidianus* larvae. This knowledge provides some implications in the conservation of this endangered animal.

## Figures and Tables

**Table 1:** Nutrient compositions of worm and fish diet. Values are presented as mean ± SE.

Nutrients	Worm Diet (g/100 g)	Fish Diet (g/100 g)
Water	90.01 ± 0.3	69.61 ± 0.6
Lipids	1.21 ± 0.1	8.66 ± 0.8
Proteins	5.11 ± 0.2	17.70 ± 0.3
Carbohydrates	1.65 ± 0.2	0.39 ± 0.0

**Table 2:** Influences of temperature and diet acclimation on metabolic rate. The differences between groups were analyzed with ANCOVA, with test temperature as covariant.

Factors	Type III Sum of Square	df	Mean Square	F Value	Sig.
Acclimation temperature (AT)	1023.872	2	511.936	10.987	0.002
Test temperature (TT)	12,955.612	1	12,955.612	278.041	<0.001
Diet	21.827	1	21.827	0.468	0.508
AT × Diet	199.824	2	99.912	2.144	0.164

## Data Availability

The datasets presented in this study can be found in online repositories (https://figshare.com/articles/dataset/Respiration_rate_and_thermal_limits_of_acclimated_Andrias_davidianus/19188443 accessed on 17 February 2022).

## Supplementary Materials

The following supporting information can be downloaded at: https://www.mdpi.com/article/10.3390/ani12040531/s1, Table S1: Influences of temperature and diet acclimation on growth rate, Figure S1: Body weight at the end of the acclimation program.
