# Supplementary material for: Temperature and Diet Acclimation Modify the Acute Thermal Performance of the Largest Extant Amphibian
Source: Animals (Basel). 2022 Feb 21;12(4):531. doi: 10.3390/ani12040531 (PMC8868240; doi:10.3390/ani12040531)
Supplement: Supplementary file 1 [file animals-12-00531-s001.zip › animals-1570663-supplementary.pdf]

Table S1. Influences of temperature and diet acclimation on growth rate. The differences between groups were analyzed with two-way ANOVA.

Factors	Type III Sum of square	df	Mean square	F value	Sig.
**Diet**	5.894	1	5.894	40.676	**0.000**
**Acclimation temperature**	5.899	2	2.950	20.356	**0.000**
**Interaction**	1.649	2	0.825	5.692	**0.005**

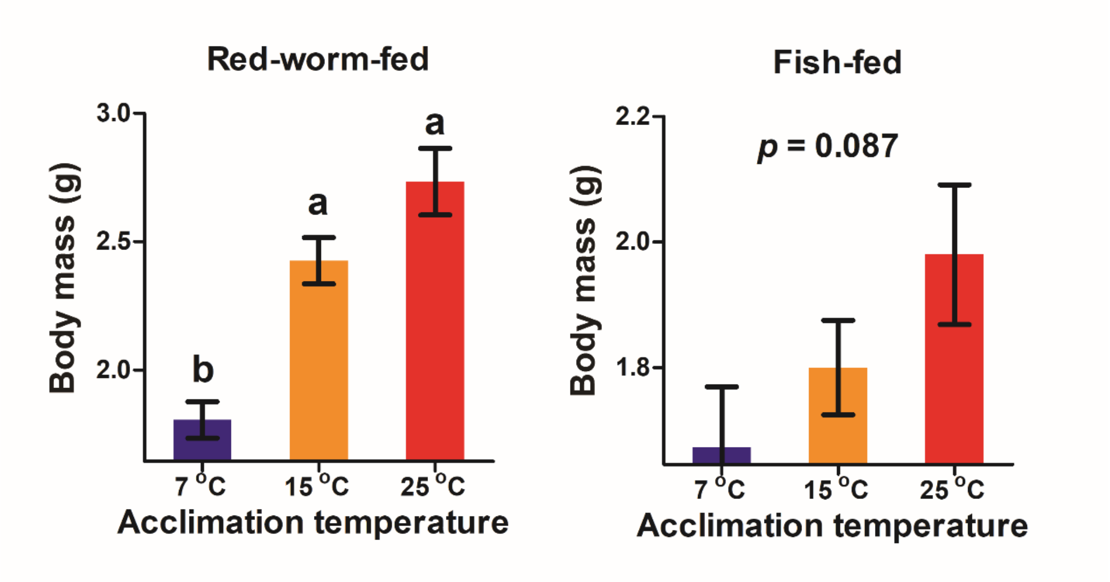

Figure S1. Body weight at the end of acclimation program. The horizontal axis denotes the initial body weight before acclimation. Different letters denote significant differences between groups, and the data was analyzed by two-way ANOVA and simple effect test.
